# Current Technical Approaches for the Early Detection of Foodborne Pathogens: Challenges and Opportunities

**DOI:** 10.3390/ijms18102078

**Published:** 2017-09-30

**Authors:** Il-Hoon Cho, Seockmo Ku

**Affiliations:** 1 Department of Biomedical Laboratory Science, College of Health Science, Eulji University, Seongnam 461-713, Korea; ihcho@eulji.ac.kr; 2 Fermentation Science Program, School of Agribusiness and Agriscience, College of Basic and Applied Sciences, Middle Tennessee State University, Murfreesboro, TN 37132, USA

**Keywords:** foodborne illness, pathogen detection, nanotechnology, biosensor, high sensitivity, rapid detection, crossflow filtration, early detection

## Abstract

The development of novel and high-tech solutions for rapid, accurate, and non-laborious microbial detection methods is imperative to improve the global food supply. Such solutions have begun to address the need for microbial detection that is faster and more sensitive than existing methodologies (e.g., classic culture enrichment methods). Multiple reviews report the technical functions and structures of conventional microbial detection tools. These tools, used to detect pathogens in food and food homogenates, were designed via qualitative analysis methods. The inherent disadvantage of these analytical methods is the necessity for specimen preparation, which is a time-consuming process. While some literature describes the challenges and opportunities to overcome the technical issues related to food industry legal guidelines, there is a lack of reviews of the current trials to overcome technological limitations related to sample preparation and microbial detection via nano and micro technologies. In this review, we primarily explore current analytical technologies, including metallic and magnetic nanomaterials, optics, electrochemistry, and spectroscopy. These techniques rely on the early detection of pathogens via enhanced analytical sensitivity and specificity. In order to introduce the potential combination and comparative analysis of various advanced methods, we also reference a novel sample preparation protocol that uses microbial concentration and recovery technologies. This technology has the potential to expedite the pre-enrichment step that precedes the detection process.

## 1. Introduction

Foodborne infection due to microbial contamination (a.k.a. foodborne disease/foodborne illness) still occurs in developed nations, and is considered a public health issue. According to the U. S. Food and Drug Administration [1], there are about 48 million reported cases of foodborne diseases in the United States annually. The strict requirements of U.S. food chains and networks are regarded as the safest in the world; however, one in six people suffer from contaminated foods or beverages in the U.S. each year [2]. Developing countries often underreport these cases. It is difficult to assess the exact global mortality and incidence of foodborne illness. Various nonprofit and regulatory organizations report growing numbers of foodborne illness [3]. According to a 2015 global estimate from The World Health Organization [4], the major causes of foodborne disease were diarrheal and invasive disease agents (i.e., *Campylobacter* spp., non-typhoidal *Salmonella enterica*, and norovirus), which resulted in 230,000 deaths (with a 95% uncertainty interval for 160,000–320,000).

The common names of foodborne illness agents are *B. cereus* food poisoning (*Bacillus cereus*), Campylobacteriosis (*Campylobacter jejuni*), Botulism (*Clostridium botulinum*), Perfringens food poisoning (*Clostridium perfringens*), intestinal cryptosporidiosis (*Cryptosporidium*), Cyclosporiasis (*Cyclospora cayetanensis*), Hemorrhagic colitis (*Escherichia coli* O157:H7), Hepatitis (Hepatitis A), Listeriosis (*Listeria monocytogenes*), viral gastroenteritis (Noroviruses), Salmonellosis (*Salmonella*), Staphylococcal food poisoning (*Staphylococcus aureus*), *Vibrio* *parahaemolyticus* (*V. parahaem olyticus*), and *Vibrio vulnificus* infection (*V. vulnificus*) [1]. It is of the utmost importance to examine food samples for the existence of pathogenic bacteria, ensure consumer health, and reduce the incidence of these food-borne illnesses. 

In general, conventional detection methods—viable plate counts, for example—have been regarded as the “gold standard”. Plating methods can detect and identify target bacteria [5] by employing non-selective and/or selective enrichments of food samples, even with low cell numbers. Time to detection is one of the most crucial limitations of traditional culture enrichment methods. For instance, conventional sample processing and plate culture-mediated analysis may require several days to obtain the final results [6], while the actual detection methods are completed within hours. This is problematic for both industry and regulatory agencies alike. Products with a shorter shelf-life may be subject to spoilage before test results are available. Pathogen detection requires a heavy workload, and creates a major bottleneck in quality control food safety laboratories. There is a universal need among food consumers, producers, processors, and researchers to develop bacterial detection methods that are fast, accurate, and easy-to-use. The significance of this public health issue is highlighted in a recent report on the global occurrences of food contamination due to *E. coli*, *Clostridium*, *Salmonella*, *Listeria*, *Cyclospora*, and *Vibrio* spp. (Table 1).

Multiple technologies are being conventionally used in concert to develop rapid and accurate cell detection methods to qualify food products. Facilitating a wider range of downstream assays using molecular biology tools (e.g., quantitative polymerase chain reaction (qPCR) and conventional polymerase chain reaction (PCR)) can increase microbial signals related to low initial numbers of target bacteria in food. Immunological methods (e.g., enzyme-linked immunosorbent assay (ELISA) and lateral flow immunoassay) are also commonly combined with traditional laboratory methodologies for the detection of pathogens in food samples. Recent pathogen detection efforts have mostly been focused on the advancement of biosensor technology. These protocols utilize various nanotechnologies—nanomaterials (e.g., metallic- and magnetic-nanoparticles) and spectroscopic methodologies, in particular—to circumvent the aforementioned detection timeframe issues. The following items should also be considered in the construction of an ideal pathogen biosensor platform: (i) high sensitivity; (ii) specificity; (iii) ease of use and interpretation; (iv) multiplex assays; (v) quantification; (vi) rapid analysis; and (vii) cost effectiveness. This review primarily focuses on the recent trends in comprehensive studies of rapid-pathogen detecting biosensors including nanostructures, nanomaterials, optics, electrochemistry, and spectroscopy. For a more practical perspective, electrochemical and lateral-flow chromatographic methods are discussed. We discuss the advantages and potential limitations of these technologies, and suggest next steps for future consideration.

## 2. Nanostructure-Based Approaches for Advanced Biosensors

According to Fan and Tamiya [24], the term biosensor can be defined as “a type of device that transduces biological recognition reactions to various physical signals including optical, electrochemical, acoustic and mechanical”. This term can also be defined as “analytical devices incorporating a biological material, a biologically derived material or a biomimic intimately associated with or integrated within a physicochemical transducer or transducing microsystem, which may be optical, electrochemical, thermometric, piezoelectric, magnetic or micromechanical” [25]. Biosensors are an increasingly powerful tool for the physicochemical detection of multiple analytes. Their specificity, convenience, low production cost, ease of use, and real-time/near real-time monitoring make them useful for a wide range of applications in clinical, food, environmental, and agricultural industries.

The recent nanotechnology boom promises nanostructures of various materials, sizes, and shapes. They have extraordinary physical properties (e.g., high surface-to-volume ratios and diffusion rates) [26] which provide diverse routes to improving pathogen detection. Nanostructures can provide promising solid substrates and signal tracers to enhance the biosensor detection of target molecules. In past decades, various optical, electrochemical, and magnetic detection methods have been combined with nanostructures. These methods were introduced to detect the harmful pathogens that cause foodborne illness. Integrated with other analytical technologies, nanostructure-based methodologies enable the rapid and sensitive detection of target pathogens, and do not involve complex sample handling. Nanostructures are extremely versatile and are widely applicable via the following optical, electrochemical, and spectroscopic approaches: infrared fluorescence, surface plasmon resonance (SPR), surface-enhanced Raman spectroscopy (SERS), and mass and nuclear magnetic resonance (NMR). Nanostructures are therefore extremely versatile.

Until these recent developments, metal-based nanostructures (e.g., Au, Ag, and Fe) have been primarily used to detect pathogens. This is due to their biocompatibility, signal enhancement effects, and ease of control. In nanobiosensor fabrication, biomolecules such as antibodies, lectins, and other binding proteins are conjugated with the nanoparticles to enable the capture of a specific target pathogen. The nanostructures and signal amplification molecules, such as horseradish peroxidase or catalase, can then be modified for enhanced sensitivity. The biosensor itself can produce magnetic signals and perceivable colorimetric resultant, which can be performed alongside the nanostructures (e.g., Au nanoparticles). Furthermore, nanostructure-based methods are practical and ready for onsite testing. They can be combined with microfluidic and membrane-based lateral-flow technologies to expedite point-of-care measurements.

Employing nanostructural materials on glass, polystyrene, and gold substrates for the immobilization of a capture binder (e.g., antibody) can also be considered as a means of signal enhancement. Choosing an appropriate material, along with the proper surface modifications and substrate patterning, is crucial for analytical sensitivity in particular. Many attempts have been made to improve the electrochemical properties of conventional and screen-printed carbon electrodes with various novel nanomaterials [27]. Such nanomaterials must meet the following requirements for enhancing electrical signals: electro-catalytic, electron transfer ability, and biocompatibility with biological molecules such as antibody capture. Such nanomaterials should provide a large surface area to increase the loading capacity and mass transport of molecules in the reaction, which results in synergic contributions to the signal amplification. The following materials are possible candidates for the supporting matrix: carbon nanotube, graphene, and nanowire, which will be described in the electrochemistry section.

## 3. Colorimetric Methods

**Figure 1:**
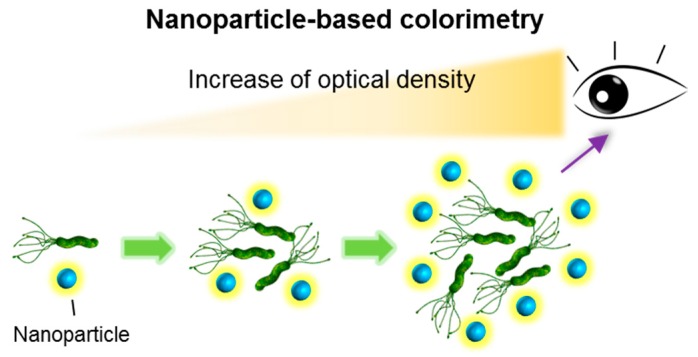
Schematic for the colorimetic detection of pathogenic bacteria in buffer and/or food suspensions using nanoparticles. Nanoparticles coated with antibody molecules capture target pathogens. As the number of particles increase, the nanoparticles’s optical signal can be enhanced in proportion to the nanoparticles. Increased colorimetric signals can be detected using the operator’s naked eye.

Colorimetric pathogen detection is regarded as one of the easiest and most practical detection techniques. Using colorimetric detection tools, presence or absence of microroganisms can usually be assessed by the naked eye, or a simple ultraviolet is spectrophotometer (Figure 1) [28]. 

Nanoparticles have become a focus of the food safety community because they can mimic the catalytic activity of biological catalysts (i.e., enzymes). Moreover, researchers have constructed a high-performance analytical system for the appropriate nanoparticle modifications. According to Jia et al. [29], some nanoparticles—CeO_2_, Au@Pt, Pt, Pd@Ir, 4-mercaptophenylboronic acid (MPBA)-modified Au@Pt nanoparticles, and Fe_3_O_4_—possess intrinsic peroxidase-like activity (Fenton’s reaction). This is analogous to horseradish peroxidase, which is commonly used in immunoassays, such as ELISA. Specifically, colorimetric signals using chromogen, such as tetramethylbenzidine, can be generated by several metallic nanoparticles without enzymatic effects (i.e., peroxidase). Enzymes and/or pretentious materials can be easily influenced by environmental conditions (e.g., temperature and pH) so there are multiple advantages of using metallic nanoparticles in the biological reaction. Moreover, unknown enzyme inhibitors may exist in the sample media; therefore, nanoparticles can be used rather than enzymes to minimize their physicochemical effects.

However, this technical approach raises a question: can colorimetric assays using nanoparticles in a complex food matrix achieve rapid microbial detection? The answer is crucial to the practical value and utility of the colorimetric assay. Multiple reports maintain that rapid detection achieved by a combination of nanoparticles and colorimetric assay result in increased sensitivity. The practical implementation of the colorimetric assay using a complex food matrix of multiple inhibitors (i.e., high level of protein, fat, carbohydrate, red blood cell, and phenolic compounds) has yet to be verified. Various colorimetric assays for microbial detection employ artificial spiking of the target cells in relatively clean and less complex solutions than complex food homogenates. This experimental method imposes significant restrictions on rapid and simple methods for existing food pathogen quantification and detection techniques with real food samples. Biosensor specificity and sensitivity can be significantly affected by the composition of food matrices. This may be caused by enzyme inhibition (e.g., competitive or non-competitive), non-specific adsorption resulting in a false-positive signal, and the interruption of antigen-antibody interaction due to the molecular components of food samples. 

Au bimetallic alloy nanoparticles were recently utilized in immunoassays [30] and glucose assays [31]. After employing the biomimetic catalytic activity of the Au nanoparticle, Su et al. [32] reported a 40 min *E. coli* O157:H7 detection protocol for cell concentrations from 7–6.8 log CFU/mL. Nanoparticle surfaces were modified into a cationic state, and the negatively-charged *E. coli* O157:H7 became electrostatically coupled. β-galactosidase was also coupled to cationic Au nanoparticles and functionalized with amine head groups to act as an enzyme-nanoparticle biosensor for bacteria detection [33]. When *E. coli* XL1, whose surface has a negative charge, binds to the cationic nanoparticle, β-galactosidase can be freed and enzymatic activity is restored. Using the field-friendly test strip format, *E. coli* XL1 suspended in phosphate buffer could be detected at the level of 2 log CFU/mL and 4 log CFU/mL within 5 min. The substrate used for β-galactosidase was chlorophenol red β-d-galactopyranoside. A chromogenic reaction occurs once the galactose moiety is cleaved by β-galactosidase. Qiu et al. [34] reported that the lysozyme-capped Au nanoparticle’s colorimetric sensing system enabled the naked-eye detection of about 3.7 log CFU/mL of *Bacillus subtilis* suspended inside a 3-(*N*-morpholino)-propanesulfonic acid (MOPS) buffer. They also concluded that their colorimetric sensor is applicable for the rapid and specific microbial detection assay of complex samples. In their methodology, the lysozyme-capped gold nanoparticles are initially purple in color due to aggregation. However, when *Bacillus* is present, the lysozyme is separated from the gold nanoparticles and attaches to *Bacillus* since lysozyme is very sensitive to bacteria, which results in the changing color of the gold nanoparticle from purple to purplish red. 

**Figure 2:**
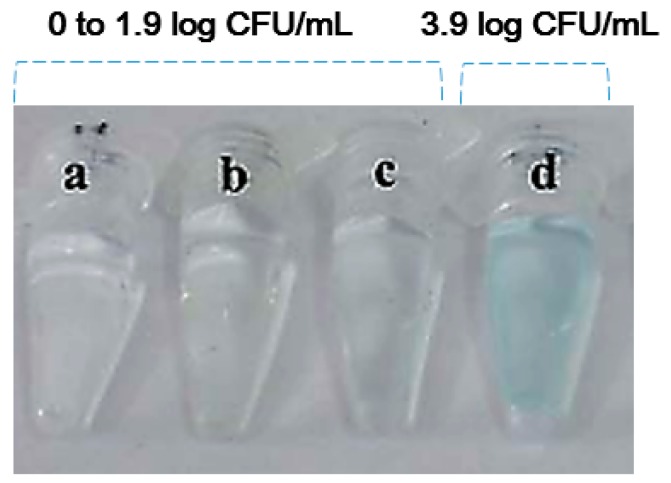
Absorption-profile of AuPt-adsorbed *E. coli* O157:H7 in the absence of bacteria (transparent, no color changes, (**a**)), due to the addition of 0.8 (**b**), 1.9 (**c**), and 3.9 (**d**) log CFU/mL of *E. coli* O157:H7. Adopted and reproduced from Su et al. [32] with permission from the Royal Society of Chemistry. A similar detection limit (>2 log CFU/mL) was also observed by Miranda et al. [33].

Thavanathan et al. [35] used different strategies to identify and quantify *Salmonella enterica* from food homogenates. The colorimetric biosensing of the *invA* gene was performed, and the technology provided a novel way to detect *invA* via direct visualization. In this work. The primary DNA probes are bound to the gold nanoparticle and secondary DNA probes that have a partial complementary sequence are immobilized on the graphene oxide. Upon introducing the *invA* gene in the solution, the two probes bind to the *invA* in a sandwich form and then the gold nanoparticles become aggregated (purple color). This *Salmonella* detection tool replaced the gel electrophoresis and had an expected cost of 25 cents per unit. This detection method is rapid, convenient, highly sensitive, and does not require an expensive signal analyzer for pathogenic bacteria detection with water or clean buffer samples. Moreover, a few measures will improve its specificity since the detection is dependent on capturing binders, like antibodies. With the appropriate optimization of the experimental procedures and protocols, it may be possible to achieve even lower limits of detection with the use of highly-sensitive optical absorption spectrometry. Although the aforementioned studies demonstrated cost effectiveness, simplicity, ease of operation, and the rapid response of the colorimetric sensor, significant weaknesses and limitations were also observed. First and foremost, the researchers [32,33,34] suspended their target microorganisms in buffer or water. These solutions are typically cleaner and less complex than food homogenates. While Thavanathan et al. [35] reported that they detected *Salmonella* in food homogenates, they did not report their protocol for producing the food homogenates. Various organizational leaders in food safety (i.e., FDA, International Organization for Standardization (ISO), Ministry of Food and Drug Safety of South Korea (MFDS), The World Health Organization (WHO), and the United States Department of Agriculture (USDA)) have clearly identified sampling methods for producing food homogenates in order to detect pathogenic bacteria in food and environmental samples (e.g., homogenating and mixing 25 g of food sample and 225 mL of buffer (e.g., buffered peptone water, lactose broth, trypticase soy broth, universal pre-enrichment broth, brilliant green water, and tetrathionate broth)). The protocol for producing food homogenates can be varied based on the specimen type and target bacteria [6,36,37,38,39]. Secondly, the utility and analytical performance of naked-eye colorimetric methods using nanoparticles is limited by the requisite high concentrations of target microorganisms (Figure 2). The detection thresholds necessary for target cells in practical field testing were more than 2 log CFU/mL in clean buffer.

These methodologies do offer handy and simple detection procedures with increased sensitivity; however, such approaches also have considerable limitations. The cell detection technologies have only been proven with artificially spiked cell samples in laboratory conditions. Further work must be done with naturally-occurring microbiota. The practical application of colorimetric assays coupled with nanotechnology for the microbial detection of real food and beverages still remains a considerable challenge due to the following possibilities: (i) the interaction between the nanoparticles and food ingredients; (ii) the uncontrollable aggregation of nanoparticles in the food matrix or food homogenates; and (iii) the presence of reaction inhibitors (e.g., protein, fat, fibers, phenolic compounds, organic acids, red blood cells, feathers, and artificial food preservatives). Recent advances in controlling nanoparticle aggregation has been characterized by the in situ immuno-Au nanoparticle network-based ELISA biosensor. These advances have improved the visualization and enhancement of colorimetric signals for microbial detection in real food samples. 

Specifically, the network approach of Au nanoparticles conjugated with antibody and peroxidase demonstrated extremely high sensitivity (3 CFU/mL for *E. coli* O157:H7 and 15 CFU/mL for *S. typhimurium*) in multiple food samples (i.e., reduced fat milk, ground beef homogenates (a mixture of 10 g of ground beef and 10 mL of sterilized phosphate-buffered saline), and pineapple juice) [40]. Direct microbial detection was carried out with magnetic separation. The overall microbial detection process was finished within 2 h of sample preparation. Switchable linkers were used to control the aggregation of Au nanoparticles, and revealed high analytical performance. The bridged nanoparticles were in proportion to the concentration of the target analyte, and less than 100 CFU/mL of *E. coli* cells in phosphate-buffered saline was detected [41]. Table 2 summarizes the experimental conditions and cell detection times for a number of Au nanoparticle studies.

## 4. Optical Methods

Fluorometric methods using various fluorescent dyes have also been used as efficient analytical means in optical sensor development. However, photobleaching and fluorescence quenching are common downsides to the use of fluorescence dyes. Recently, methodologies coupled with nanoparticles were introduced as novel optical detection methods. These approaches not only enhanced signals, but also circumvented the intrinsic disadvantage of fluorophores [42]. Quantum dots offer a competitive signal tracer and chemical stability. However, the chemical toxicity for living cells limits quantum dot usability.

**Figure 3:**
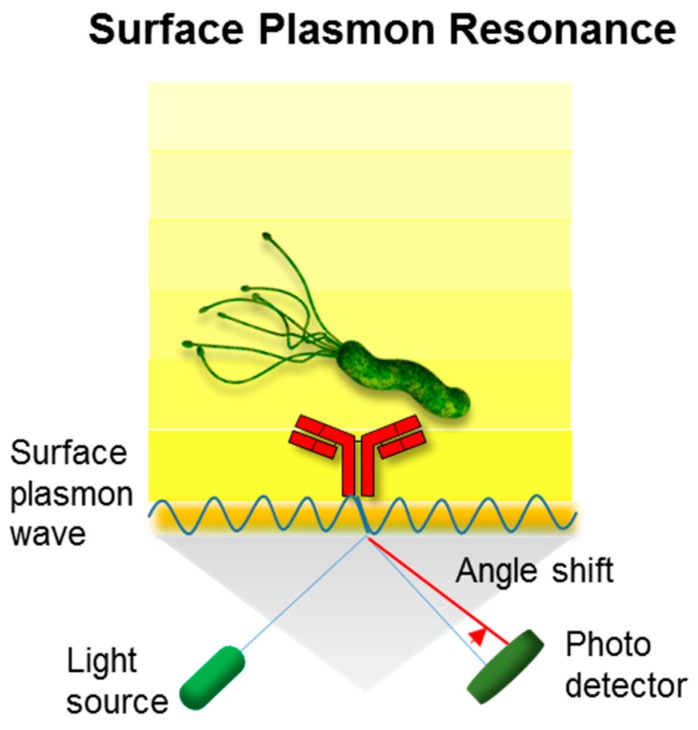
Schematic representation of the optical approach based on surface plasmon resonance (SPR). This system is performed with a light source and thin metallic material (e.g., Au). An electromagnetic light wave can be coupled with a surface plasmon wave and change the angle shift of the reflected light, which is the result of the binding between the antibody and pathogen. This detection tool can be utilized for the real-time monitoring of sample pathogens.

Given the optical property of metallic substrate, surface plasmon resonance (SPR) can be applied in pathogen detection. SPR measures the charge-density oscillation at the interface of two media (e.g., metal and dielectric). The refractive index is changed due to the binding events on the metal surface, which enables single-step, rapid, label-free, and real-time target monitoring (Figure 3) [43].

However, low analytical sensitivity can result from a small refractive index, slow diffusion-driven mass transfer, or the insufficient depth of the influenced layer. These are intrinsic problems in the conventional SPR methods as well. Instead, antibody-nanoparticle conjugates can be used as signal enhancers in sandwich complex formations to circumvent SPR’s small refractive index. The localized surface plasmon resonance (LSPR) approach can be applicable for label-free, real-time pathogen detection. Since LSPR is a phenomenon based on the interaction between a specific light wavelength and plasmonic nanoparticles, a small and cost-effective biosensor system can be constructed accordingly. Although LSPR has problems similar to SPR, especially the rapid decay of the surface plasmon, employing fragmented antibodies (e.g., Fab) or small binders (e.g., aptamer) with the proper surface modification of the nanoparticle is a possible solution [43]. 

Long-range SPR was also combined with magnetic nanoparticles to detect pathogens. The SPR propagates along the thin metal films were embedded in a refractive index symmetrical layer architecture and combined with magnetic nanoparticles and resulted in a dark-field light-scattering imaging technique associated with Au nanoparticles for *E. coli* DH5α [44,45]. Detection and/or counting in artificially-spiked water, milk, and fruit juice samples was achieved within 30 min, with recognition percentages of 77%, 73%, and 71%, respectively. This technology had a dynamic detection range of 2–4.8 log CFU/mL. While the experimental tool presented by Xu et al. [45] is valuable, it should be noted that their *E.coli* detection and/or counting systems showed significant differences in cell counts compared to manual plating methods. They clearly showed the margin of error of manual counting, yet their protocols were intended to be cost-effective. Phillips et al. [46] reported another approach to detect microorganisms using a polymer-based optical method. These researchers used “gold-nanoparticle–conjugated polymer constructs” for microbial detection; specifically, they employed fluorescent polymers, such as poly-(*para*-phenylene ethylene), that electrostatically interact with gold nanoparticles. With this technique, 20 varieties of bacteria (e.g., *E. coli*, *Lactobacillus*, *Bacillus*, *Streptomyces*, *Pseudomonas*, *Streptomyces* spp.) could be detected in phosphate buffer. During the detection process, anionic poly-(*para*-phenylene ethylene) is attached to cationic gold nanoparticles in the initial condition, which causes a quenching fluorescence signal. However, once the target bacteria are present, the gold nanoparticles attach to the cell and the polymers are released from the particle, recovering the intrinsic fluorescent signal. Though the research group demonstrated the potential use of fluorescent polymers and functional gold nanoparticles as a biodiagnostic tool, they did not clearly specify the detection limits and overall experimental time required for the recognition of their target cells. 

**Figure 4:**
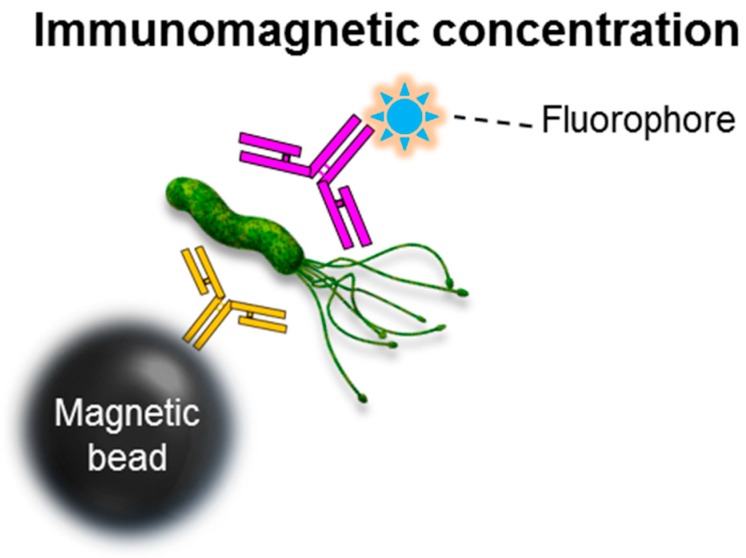
Schematic for magnetic, bead-assisted pathogen detection using fluorescence enhancement. The initial concentration of the pathogen in the food rinse or testing buffer can be increased using a simple immunomagnetic separation processes. This microbial detection tool utilizes paramagnetic beads and the antibodies specific to the target food pathogen. The magnetic bead and target cell complex can be separated from the food suspension with a magnet and transferred to a cuvette for fluorescent detection.

For the past two decades, magnetic nanoparticles have been utilized to separate and concentrate target analytes from aqueous solutions. The use of magnetic nanoparticles is still considered a simple and powerful technique, especially for sample purification via magnetic separation without complex instrumentation. According to El-Boubbou [47], there are multiple advantages to using magnetic nanoparticles: (i) large surface area that directly influences the microbial adherence efficacy of particles and surface/volume ratio; (ii) nano-sized particles that offer high levels of cell wall adherence, resulting in effective separation from the aqueous solution; and (iii) faster kinetics are possible liquid-phase reaction with the target, thereby enabling the efficient detection of a pathogen. For example, iron oxide nanoparticles have been researched as promising tracers for pathogen detection (Figure 4).

Magnetic glycol nanoparticles with functionalized D-mannose have captured *E. coli* with outstanding efficiency (i.e., 88% of capture efficiency following a 45 min incubation procedure). This technique’s detection limit was more than 4 log CFU/mL, and the overall experimental time (45 min incubation, 5 min detection) was more than 50 min [47]. When labeled with antibiotics (e.g., vancomycin, gentamicin), magnetic nanoparticles also bind to the receptors on bacterial cell walls of bacteria. Such magnetic nanoparticles were also used for the rapid and highly-efficient capture of target pathogens. Using bioactive magnetic nanoparticles (vancomycin-conjugated FePt nanoparticles), Gu et al. [48] reported the capture and detection of *Enteroccocus faecalis* (ATCC 29212) and *Staphylococcus aureus* in concentrations as low as 1 log CFU/mL within 1 h. The bio-conjugation of fluorescent magnetic nanoparticles and gentamicin (via glutaraldehyde) showed an effective adhesion to *E. coli* W3110 within 20 min in phosphate-buffered saline at a concentration of 3 log CFU/mL [49]. However, the specificity issue is an obstacle to using lectin and antibiotics. Recently, the Irudayaraj group proposed a fluorometric immunological method coupled with magnetic separation for the rapid detection of three pathogens (i.e., *E. coli* O157:H7, *S. typhimurium*, and *L. monocytogenes*) in real samples with high sensitivity which required less than 2 h without enumeration [50]. In this study, they performed an in situ immunoassay using a magnetic bead and probe for denatured bovine serum albumin labeled with fluorophores, which can be released via enzymatic digestion. They reported the limit of detection (LOD) was less than 5 CFU/mL for *E. coli* O157:H7, *S. typhimurium*, and *L. monocytogenes* in foods (from spinach, chicken, and milk, respectively). 

## 5. Magnetic Detection Methods

In recent research, NMR spectroscopy has been considered a powerful measurement tool for magnetic nanoparticle-based detection. It is used to measure the spin-spin relaxation time (*T*2) of water protons on magnetic nanoparticles-tagged bacteria samples. The performance was enhanced by synthesizing iron-based magnetic nanoparticles with high transverse relaxivity. For example, Kaittanis et al. [51] developed a one-step cell detection method using nanoparticles and magnetic relaxation. They detected *Mycobacterum avium* spp. *paratuberculosis* in milk and blood samples. Lee et al. [52] also reported core-shell nanoparticles and an NMR-filter system to detect *Bacillus* spp. within 30 min in as few as 1.3 log CFU/mL suspended in buffer solution. Although the idea presented herein is promising and useful for microbial detection, the aforementioned practical problems remain. The researched assays have been carried out in clean buffers rather than real food or biological suspensions. Food matrix-associated ingredients offer potential interference in bacterial detection procedures involving receptor and ligand interactions. Microbial detection from a real food matrix, thus, involves critical obstacles. Reducing the non-specific adsorption of food matrix components is a key consideration, which may be alleviated with a surface coating of inert chemicals and biological materials.

## 6. Electrochemical Detection Methods

The amplification strategies of electrochemical analysis can be categorized as follows: (i) employing novel materials as a supporting electrode matrix to facilitate electron transfer; or (ii) labeling approaches using bioconjugates with nanomaterials used as a carrier of electroactive tracers or enzymes. Since the electrode provides a solid support for the capture binder (e.g., antibody), as well as a sensing means for the electrons, choosing a proper electrode associated with the proper surface treatment is crucial to achieve effective analytical performance [53].

Metallic nanoparticles that possess intrinsic electronic properties (along with sufficient surface area, mechanical strength, and catalytic properties) have been used to enhance electrochemical signals. Their potential signal amplification has been guided toward pathogen detection [54]. Varshney and Li [55] used impedance biosensors to detect bacteria in phosphate buffered saline and supernatants of centrifuged ground beef homogenates (250× *g* for 15 min). These sensors incorporate interdigitated array microelectrodes with antibody-coated magnetic Fe nanoparticles. Using the principle of impedance, they could detect 4.9 log CFU/mL and 5.9 log CFU/mL of *E. coli* O157:H7 from PBS and the supernatant, respectively, within 35 min from sampling to measurement. They concluded that rapid and specific detection of *E. coli* O157:H7 in ground beef samples was achieved. Although their technology has the potential to replace the currently time-consuming microbial detection process, limitations still exist for industry applications. The conventional detection methods—including PCR, plating methods, and time consuming cultural enrichment steps—may still be necessary for the low cell detection level from the ground beef sample.

*E. coli* is a common facultative anaerobic bacterium found in the human intestinal tract. It has been used as a biomarker for the fecal contamination of foods [56], where *E. coli* O157:H7 can exist at low concentrations. According to the FDA [57], a 1–2 log CFU dose of *E. coli* O157:H7 cells can be infectious to food consumers. Therefore, detection of low levels of *E. coli* O157:H7 must be a goal for food safety research. Based on ISO, WHO, and/or FDA protocols, ground beef homogenates are produced by mixing 25 g of ground beef and 225 mL of buffered peptone water [35,37]. Varshney and Li [55] further introduced 15 min of centrifugation, which precipitated both food particles and naturally occurring microbiota in ground beef homogenates. Since only supernatants were used for microbial detection, it is possible that most cells in the food homogenates were removed during the centrifugation. Moreover, differences in the detection limits of centrifuged ground beef homogenates were detected based on the ground beef components (i.e., proteins and fats). Electrochemical detection is further limited by the low capture efficiency of the immobilized surface and the presence of conductive proteins. The detection thresholds are not satisfactory for direct industry applications; however, the microelectrode-based impedance biosensor remains a potential cell detection strategy for food systems [58,59]. 

Setterington and Alocilja [60] also reported an electrochemical biosensor for the detection of immunomagnetically-separated microorganisms. They used immuno-functionalized magnetic/polyaniline core/shell nanoparticles to separate *B. cereus* and *E. coli* O157:H7 from peptone water. Polyaniline was employed as a conducting material for electrical analysis. They combined cyclic voltammetry measurements on screen-printed carbon electrodes and immunomagnetic separation processes to increase detection performance. The detection limits for *B. cereus* and *E. coli* O157:H7 were 1.6 log CFU/mL and <1 CFU/mL, respectively. 

Afonso et al. [61] discussed a disposable immunosensor which employs a magneto-immunoassay and gold nanoparticles. This electrochemical tool was used to detect *S. typhimurium* in PBS and skim milk. Detection limits at 2.2 log CFU/mL *S. typhimurium* were reported for PBS, but the research group did not mention the exact LOD of the skim milk. Moreover, they spiked a high concentration of cells (7 log CFU/mL) into the skim milk to examine the immunoassay specificity. Afonso et al. concluded that their method is suitable for the early screening of *Salmonella* in real samples and potentially an alternative to cell identification in skimmed milk; however, critical limitations were also noted. The recovery percentage of *S. typhimurium* decreased in proportion to the reduced cell levels (from 94% to 83%) in diluted skim milk by a factor of 10 in PBS-Tween solution. Moreover, the non-specific binding of antibodies can occur, which results in a reduced recovery percentage for 100% skim milk. Nevertheless, this disposable immunosensor is a potential tool for easy-to-use cell detection. It has several possible applications in beverage and environmental industries, specifically where rapid microbial detection in clean and purified solutions is necessary.

**Figure 5:**
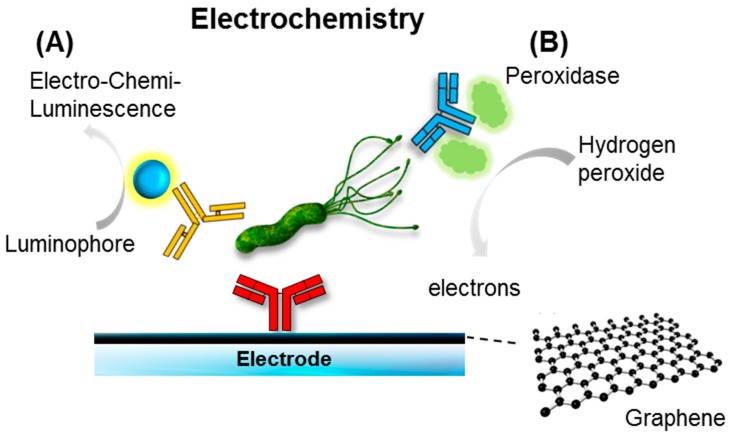
Schematic illustration of an electrochemical immunosensor; (**A**) a luminophore undergoes redox reaction in the electrodes with the co-reactant, producing photons that can be detected by the photodetector (e.g., a complementary metal–oxide–semiconductor (CMOS) and charge coupled device(CCD)); (**B**) similar to a common glucose sensing biosensor, electrons can be produced via enzyme catalytic reaction (e.g., peroxidase), which is labeled with the antibody in the presence of the substrate (e.g., hydrogen peroxide). Graphene, a carbon allotrope (in the form of honeycomb), was recently used as a supporting matrix for enhancing electrical performance.

Among the electrochemical solutions in development are carbon nanotubes, nanowire, and graphene carbon allotropes [62,63,64,65,66,67,68]. Both have proven to be very promising materials in terms of signal amplification. Both can be utilized as electrode scaffolds with outstanding chemical stability, rigidity, surface area, and conductivity [62] (Figure 5). Many attempts have been made to improve the electrochemical properties of conventional and screen-printed carbon electrodes with a variety of novel nanomaterials [63]. These nanomaterials have a massive surface area, which can also support the increased loading capacity and mass transport of the molecules participating in the reaction. These processes result in synergic contributions to signal amplification.

## 7. Raman Spectroscopic Methods

**Figure 6:**
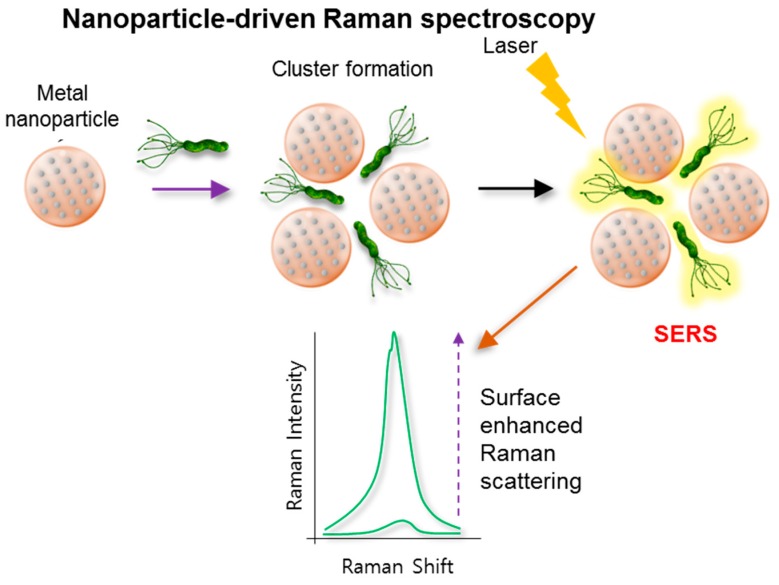
Schematic of the nanoparticle-driven Raman spectroscopic tools for the detection of the pathogen. The optical properties of nanoparticles vary when particles and target cells are agglomerated. Surface-enhanced Raman scattering (SERS) generates plasmonic nanostructures and enhances the Raman scattering of adsorbed molecules (e.g., gold nanoparticles) upon the irradiation of a specific laser wavelength, resulting in the enhanced Raman scattering of adsorbed molecules (e.g., Raman reporters). SERS efficiency is directly related to the proximal distance among the cluster’s plasmonic particles.

**Figure 7:**
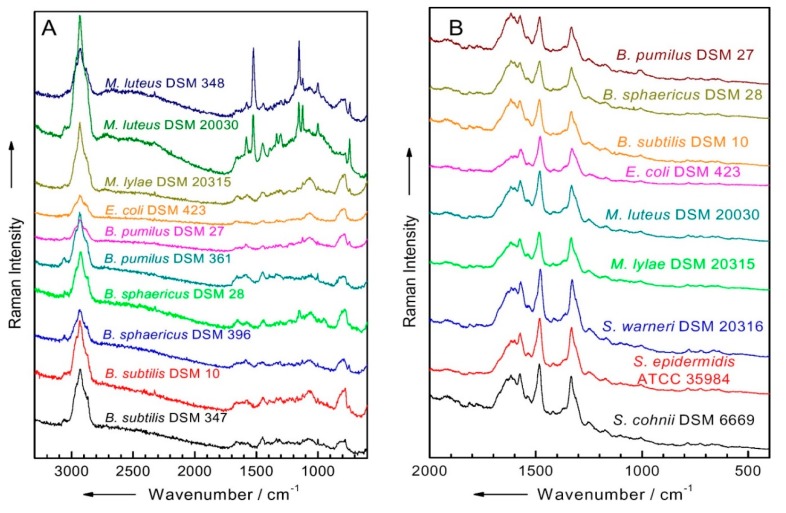
(**A**) Characterizing single microbial cells using micro-Raman spectra at 532 nm excitation. (**B**) Bulk microbial samples (up to 6 log cells) using ultraviolet-resonance Raman spectra excited at 257 nm (Adopted and reproduced from Popp [60] with permission from SPIE publication).

The Raman spectroscopic method has the potential to identify microorganisms in biological samples within hours. This technique has been widely applied in the field of pathogen detection, including various food matrices [69]. Like IR-based fingerprinting analysis, each species of microorganisms displays a unique Raman signal (Figure 6 and Figure 7) [70]. Cell signals can be increased using antigen-antibody reactants and attaching nanoparticles to cell walls or membranes. In some cases, Raman signals from a single microorganism are sufficient for bacterial identification [71]. SERS amplifies the Raman signal of molecules nearby plasmonic nanoparticles, yielding a significant 6- to 112-fold log increase [72]. A combination of Ag nanoparticles and Ag nanospheres were used to produce a “surface enhanced Raman scattering active substrate.” This resulted in increased Raman scattering into the target samples. To generate the conjugated structure of Ag nanoparticles and Ag nanospheres, a finite-difference, time-domain analysis of the electromagnetic field was used. In this work, magnetic nanoparticles demonstrated the potential to concentrate pathogens at the sensor surface. This enabled the detection of three microorganisms (i.e., *E. coli* O157:H7, *Staphylococcus aureus*, and *Salmonella typhimurium*) with a LOD of 1 log CFU/mL in phosphate buffered saline. Using a simple procedure, the gold nanoparticles associated with the silica-coated magnetic nanoparticles were also successfully used to separate and detect bacteria. Raman reporters are often comprised of thiolated compounds (e.g., mercapto-benzoic acid and mercapto-pyridine) and usually generate very strong SERS signals. When coupled with gold nanoparticles, the detection of multiple targets via SERS finger print region differences was possible. In the multiplex SERS measurement work of Wang et al. [44], 4 log CFU/mL of *S. typhimurium* and *S. aureus* were detected simultaneously. 

The SERS technique was further refined with a filter membrane (0.45 µm pore size) that can trap nanoparticle probe-labeled pathogens. This membrane-associated SERS method enables the multiplex, efficient (45 min), and sensitive detection of bacteria in PBS with a series of simple steps [73]. In this work, gold, silver, and gold–silver core–shell nanoparticles were coupled with microbial antibodies, followed by additional labeling of Raman reporter molecules. The reported LOD was in the range of 2–3 log CFU/ml. However, this work also showed two major limitations: (i) researchers suspended their target cells (i.e., *S. enteritidis*, *E. coli* O157:H7, and *S. aureus*) into clean PBS buffer; and (ii) filtered out their microbial samples using a 0.45 µm filter membrane. This is a larger pore size than conventional filter cut-offs (0.2 µm cutoff) for the microbial separation of biological suspensions.

Raman spectroscopy demands a skilled operator and considerable capital for initial equipment costs. That being said, less costly miniaturized Raman systems are available for use in the field. Spectral variances among the cell species are often observed together, so the application of biochemical tools (e.g., PCR, immunological methods) is still necessary to ensure accuracy. Even though Raman signals can be chemometrically and statistically analyzed for microbial identification [71], the use of biochemical tools is still strongly recommended by regulatory organizations. Therefore, the development of cost-effective, robust Raman spectroscopic methods should be pursued.

## 8. Mass Spectroscopic Methods

**Figure 8:**
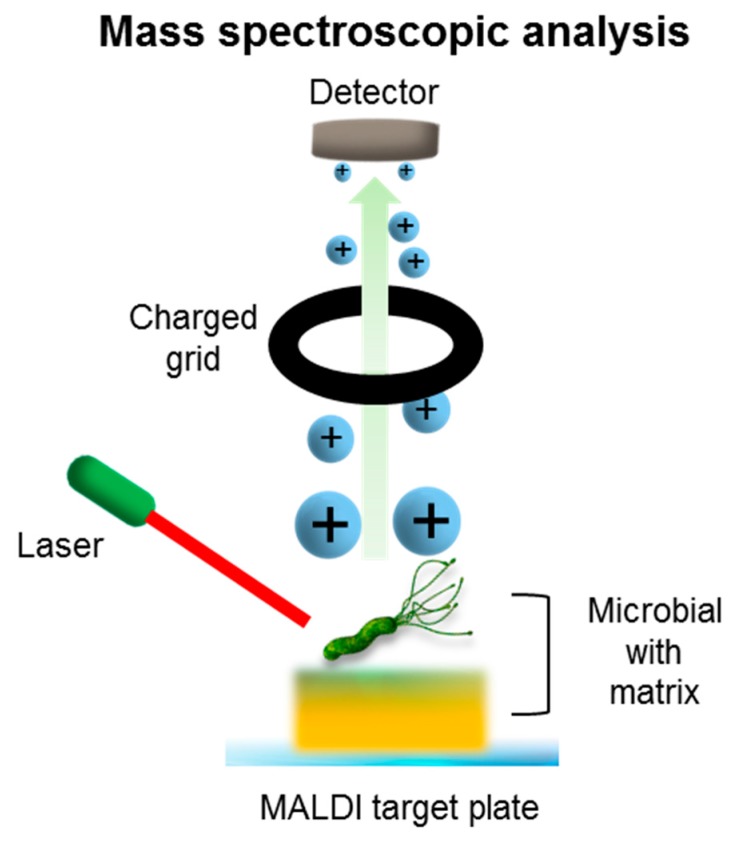
Schematic illustration of mass spectroscopic method (e.g., Matrix-assisted laser desorption/ionization time-of-flight (MALDI-TOF)) performed via laser-driven ionization of the target sample followed by time-of-flight analysis of peptide fragments for the identification of a specific pathogen.

Matrix-assisted laser desorption/ionization time-of-flight (MALDI-TOF) mass spectrometry for pathogen detection has been reported (Figure 8). With its proteome profiling property and ability to add new organisms, the MALDI-TOF technique enabled the identification and analysis of various target bacteria [74]. The key weaknesses of MALDI-TOF are: (i) high initial set-up costs; (ii) the necessity of enrichment growth prior to testing; and (iii) the need for peptide mass fingerprints in the spectral database to identify the microbial species and strains that act as control markers.

Seng et al. [75] has applied MALDI-TOF for clinical purposes. Rather than food samples, target microbial colonies were obtained from blood, cerebrospinal fluid, pus, biopsies, the respiratory tract, wounds, and fecal specimen. They were then deposited on MALDI-TOF plates. Microbial detection of the plates took 8.5 min per sample (i.e., 2 min for colony deposition on the plate, 2 min for air-drying the matrix-sample crystallization, 0.5 min for spectra acquisition, and 1 min for cell identification of spectra).

However, analytical sensitivity has been a major drawback of mass spectroscopy-based measurements due to low levels of specimen target cells. Various nanoparticles (Au, Pt, TiO_2_, Se, CdTe, Fe_3_O_4_, and Pt) were employed to increase this sensitivity. Metal nanoparticles represent light-scattering characteristics in the visible region, and work similarly to the aforementioned microbial detection tools [76]. The coating of numerous nanoparticles on the bacterial cell facilitates ionization. Recently, antibody-functionalized Pt nanosensors were used to identify plant-associated bacteria (i.e., *B. thuringiensis* and *B. subtilis*) in soil and carrot samples. As a result, the bacterial protein marker signals were enhanced during the MALDI assay [77]. Although biological and environmental samples were used, this work did not evaluate the detection limits or the overall time frame for microbial cell detection. The Ahmad research group also did not include the sample solution processing methods for routine food industry use. Further studies that consider industrial applications and identify food-associated bacteria within complex food matrices are needed.

## 9. Lateral-Flow Chromatographic Assay Methods

**Figure 9:**
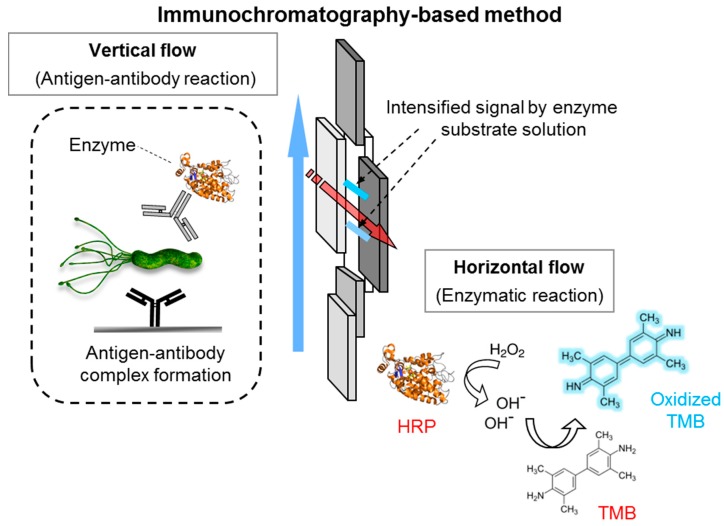
Schematic illustration of colorimetric analytical method associated with enzyme-based immunochromatography (e.g., horseradish peroxidase) for onsite testing. Consists of sequential vertical and horizontal flows of the antigen-antibody reaction and enzymatic color signal generation, respectively. The signals (e.g., oxidized tetramethylbenzidin) adsorbed on the membrane pad can be analyzed qualitatively and quantitatively.

**Figure 10:**
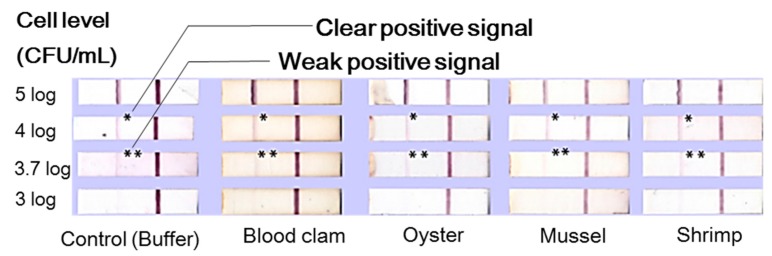
Detection of *Vibrio cholera* O139 using a lateral-flow, immunochromatographic strip conjugated to a gold nanoparticle. Adopted and reproduced from Pengsuk et al. [84] with permission from Elsevier.

Strip-based lateral flow chromatographic assays (LFA) are one of the most practical, simple, and widely-used methods for the detection of target analytes (e.g., whole cells, antibiotics, toxins) in sample matrices. These techniques have no need for expensive instrumentation [40,78,79,80,81,82,83,84,85,86,87]. In principle, LFA uses a porous membrane strip as a solid substrate to immobilize and capture ligands (e.g., antibodies or aptamers that bind to a specific target). This is a powerful technique for detecting analytes in samples like blood, urine, and saliva. Typical LFA setup components are depicted in Figure 9. Gold nanoparticle tracers have large coefficients of extinction; therefore, the color of these nanoparticles can be easily recognized by the naked eye. The LFA antibody-antigen interaction can be performed in a single step. Rapid time to results and ease of use increase LFA’s utility in point-of-care diagnostics. Gold nanoparticle coupled with *Vibrio cholera* 0139-specific antibodies detected target bacteria in seafood (i.e., blood clam, oyster, mussel, and shrimp) homogenates at an LOD of 4 log CFU/mL [84]. The research group also observed weak, positive signals at 3.7 log CFU/mL (Figure 10).

To date, most LFA formats for pathogen detection are based on color signals from gold nanoparticle tracers perceivable to the naked eye. These LFA formats exhibit sensitivities significantly lower than conventional immunological methods (e.g., ELISA). The current LOD for a gold tracer-based LFA is approximately 4–6 log CFU /mL. The early stage assessment of target bacteria is not possible due to these small, initial concentrations. Pre-enrichment processes must be incorporated to proliferate the initial target bacteria. To date, a series of modified LFA methods combined with nanotechnology to enhance analytical sensitivity have been reported. Cho et al. developed a cross-flow chromatography that enables ELISA signal generation to the membrane [85]. Rapid detection of *L. monocytogenes* in complex matrices was accomplished via immuno-magnetic separation coupled with lateral flow enzyme immune-concentration. The chromatographic analysis yielded a LOD of <2 log CFU/mL in both the buffer solution and 2% milk sample [80]. The signals can be significantly amplified with the use of gold nanoparticles in combination with enzymes. In this case, the nanoparticles act as an enzyme carrier. Since the signals obtained from the assay are directly proportional to the amount of enzyme participating in the reaction, it is crucial to tether as many HRP molecules to gold nanoparticles as possible to obtain a higher sensitivity [80]. 

The two conjugates designed for this purpose are a biotinylated gold nanoparticle and a streptavidin-horseradish peroxidase construct. Due to the nanoparticles’ large surface areas, SA-HRP conjugates can be efficiently bound to gold nanoparticles via strong avidin-biotin interactions (molecular affinity is about-15 log mol/L) with minimal steric hindrance. The LOD could be enhanced up to 1000-fold compared to the nanoparticle-based commercialized kits with this simple modification alone. Liposome can also be applied to the LFA for signal enhancement. Ho et al. [86] prepared immuno-liposomes that contained methyl blue dyes to detect heat-killed *Salmonella typhimurium* (1.68 × 10^3^ cells/mL). Shulkla et al. [87] also demonstrated a liposome-based approach with reverse-phase evaporation that resulted in 2 log CFU/mL of *Salmonella* typhimurium. Geometric systems changes may also have an influence on the analytical performance of LFA. Lutz et al. [88] controlled the speed of the sample flow by applying different concentrations of sugar, therefore, making multiple steps towards simple malaria detection without instruments.

Lateral flow immunochromatographic assays that use nanoparticles as signal tracers feature significant advantages. These methods report results (i.e., the presence or absence of target cells) very quickly (about <20 min). They are simple, highly-sensitive, selective, low-cost, and versatile, especially compared to other methodologies. However, as summarized by Posthuma-Trumpie [89] and Sajid [90], multiple restrictions and weaknesses of lateral flow immunoassays still exist (Table 3). These drawbacks can be properly circumvented by slight modifications of the current lateral-flow methodologies as follows: the modulation of the membrane pore size, the concentration, and purification of the sample via magnetic separation, and the additional flow step for enzyme-based signal amplification and quantification by incorporating a semiconductor-based imaging system.

## 10. Pre-Treatment: Cell Separation, Concentration, and Recovery from Foods

Despite the progress in microbial detection technology, some detection methods still have weaknesses in detecting low-level pathogens present in food samples suspected of contamination. Obviously, many research groups have shown remarkable results in the rapid detection of food pathogens using water, juice, and/or buffers containing significantly lower levels of detection inhibitors compared to food or food homogenates. Inhibitors present in foods adversely affect the sensitivity of microorganism detection. This technical hurdle has been addressed with pre-enrichment, which increases the total number of cells in the food sample. As we have previously mentioned, the pre-enrichment process can be considered as a major obstacle to high-speed microbial detection protocols. In actual microbiological assays, the identification process can be completed within a few hours. However, due to the utilization of a time-consuming pre-enrichment process, the actual microbial detection time is usually greater than the actual detection time. Table 4 summarizes factors worth consideration to develop the ideal pre-treatment method for the detection of microorganisms.

Several groups have focused on the development of novel strategies to reduce the lengthy pre-enrichment process by microbial concentration. Among them, the Ladisch group has developed systematic approaches to decrease lengthy microbial enrichment times [91,92,93,94,95,96,97,98,99]. They recently won the Grand Prize at the FDA Competition for their rapid *Salmonella* detection in foods. They described how filter membranes (e.g., flat sheet and cross-flow filters) and enzyme treatment affect the recovery of pathogenic bacteria in food (i.e., egg whites [94,95]) and food homogenates (i.e., ground turkey, spinach, chicken carcass, and chicken leg) [96,97,98,99]. Their protocols consist of: (i) mechanical microbial fragmentation within the food matrix (e.g., stomaching, rinsing, or blending); (ii) pre-filtration (i.e., borosilicate glass microfiber and nylon net filter); (iii) enzymatic degradation of food particles; (iv) cross-flow microfiltration using hollow fiber filter modules and microfugation; and (v) target cell detection using plating, conventional PCR, or commercial kits (Standard BAX^®^ System Assay) [92,96,99]. The Ladisch group integrated hollow fiber filter modules (polyethersulfone, 0.2 µm cut-off) into components of the automated Continuous Cell Concentration Device (C3D). Their operational process for sample concentration, recovery, and washing is automated through a graphical interface designed by LabVIEW System Design Software (National Instruments^TM^, Austin, TX, USA) [99]. The C3D system concentrates food samples to accelerate the detection of food borne pathogens. The C3D processing sequence consists of: (i) enzyme treating the food concentrations using hollow fiber microfiltration; (ii) recovery of the concentrated sample; and (iii) sterilizing the C3D system using water, 0.2 M sodium hydroxide, and 70% ethanol [97]. 

Existing microfilter-based food pretreatment equipment has several limitations. Chiefly, these expensive components are difficult to reuse. The disposable nature of existing filters, therefore, proves a significant problem, as the unit prices are very expensive. However, Ku et al. [94] has shown that the hollow fiber microfilter module can be reused up to approximately 23 times. This is facilitated by sterilization and cleaning procedures following each sample concentration. Reuse has greatly reduced the operating costs. The automated C3D system accelerates sample preparation by enabling rapid microfiltration while controlling the membrane fouling and maintaining a constant rate of flux and linear velocity [94]. As mentioned earlier, pre-filtration used to be performed to remove large food particles and prevent clogging of the filter module inlet before microfiltration [96,97,98]. Though pre-filtration efficiently removes large particulates, it also has the unintended effect of trapping microorganisms. This constitutes a major fraction of the microorganisms initially present in the sample. According to Ku et al. [96], The small particulates responsible for this effect were formed by the endopeptidase hydrolysis of meat extract, which decreased the particle size from 100 to 1 μm. The small particles then became trapped in the membrane and prevented the passage of microorganisms. The Ladisch group minimized the loss of viable sample microorganisms by performing pre-filtration before enzyme hydrolysis. This resulted in more than 80% recovery of the viable microorganisms initially present. When filtrates are treated with endopeptidase, concentrated via hollow fiber microfiltration, and microcentrifuged, the detection of 1 to 10 CFU *Salmonella*/g or mL of food samples was achieved in <8 h [94,95,96,97,98,99]. Their goal was to avoid the time-consuming pre-enrichment stage and provide alternative ways to existing qualitative analysis tools. This variety of rapid methods, including improved techniques for the detection of target pathogen and novel techniques for microbial separation and concentration, can facilitate rapid microbial detection from foods. 

## 11. Conclusions

Academia has recently turned its intention to various advanced nanomaterial technologies, optics, and spectroscopy, coupled with biosensors for rapid microbial detection. We summarized the multidisciplinary, technology-driven solutions for pathogen detection from foods. Each method has its own intrinsic advantages that improve the analytical sensitivity and the potential for onsite sensing. Despite advances in analytical technologies, pre-culture and/or pre-treatment of the enrichment media are still necessary before proceeding to an actual qualitative analysis due to the limited sensitivity or inhibitors present in the food samples. Although many reports claim rapid detection of low doses of pathogens, most have only reported the actual detection time and often lack a clear description of the preceding processes. Before detection, it is necessary to tailor the appropriate pretreatment protocols for the inherent characteristics of particular food types. Above all, sample preparation is vitally important, and its complexity is a universal challenge among all detection systems. The pretreatment stage is the rate-limiting step in current biosensing technologies. With further advances in sample preparation methods, nano/micro technology-based biosensors will offer specific, cost-effective, and robust pathogen detection. The combination of various advanced methods is promising, and some research groups are attempting to combine new sample preparation methods and advanced microbial detection methods to minimize the inherent drawbacks of some analytical methods. These platforms will be used for onsite testing and routine applications to ensure safety standards across industries. Future research must respond to these unique obstacles and achieve comprehensive detection. Additionally, some crucial analytical factors—accuracy, precision, quantitation, and validation—should be considered to achieve a reliable detection method. 

## Figures and Tables

**Table 1:** Selected 2016 global foodborne outbreaks by food carrier.

Food	Pathogen	Country	Reference
Beansprouts	*S. Saintpaul*	Australia	SA Health ^1^ [7]
Processed foods	*Listeria monocytogenes*	Canada	CFIA ^2^ [8]
N/S^4^	*Salmonella* spp.	New Zealand	PHS ^3^ [9]
Milk	*E. coli*	Romania	EFSA ^5^ [10]
Sliced chicken fillet	*Listeria monocytogenes*	France	RASFF ^6^ [11]
Smoked salmon	*Listeria monocytogenes*	Germany	RASFF [12]
Shrimp Sushi	*Vibrio cholerae*	Norway	RASFF [13]
Chicken breast	*Salmonella enteritidis*	Czech Republic	RASFF [14]
Unknown	*Listeria monocytogenes*	Italy	Eurosurveillance [15]
Mixed salad	*E. coli*	UK	WHO ^7^ [16]
Frozen Vegetables	*Listeria monocytogenes*	USA	CDC ^8^ [17]
Flour	*E. coli*	USA	CDC [18]
Raw milk	*Listeria monocytogenes*	USA	CDC [19]
Pistachios	*Salmonella Montevideo*	USA	CDC [20]
Alfalfa sprouts	*Salmonella spp.*	USA	CDC [21]
Packaged salads	*Listeria monocytogenes*	USA	CDC [22]
N/S	*Clostridium perfringens*	Korea	MFDS ^9^ [23]
N/S	*E. coli*	Korea	MFDS [23]
N/S	Norovirus	Korea	MFDS [23]

^1^ SA Health denotes the South Australian Health of Government of South Australia. ^2^ CFIA denotes the Canadian Food Inspection Agency. ^3^ PFS denotes the Public Health Surveillance Information for New Zealand Public Health Action. ^4^ N/S denotes not specified. ^5^ EFSA denotes the European Food Safety Authority. ^6^ RASFF denotes the Rapid Alert System for Food and Feed. ^7^ WHO denotes the World Health Organization. ^8^ CDC denotes the Center for Disease Control and Prevention. ^9^ MFDS denotes the Ministry of Food and Drug Safety of South Korea.

**Table 2:** Representative research on colorimetric assays coupled with Au nanoparticles for pathogenic bacteria detection.

Target Microorganism	Detection Limit (CFU/mL)	Detection Time (min)	Aqueous Solution	References
*E. coli* O157:H7	<7 log	40	Water	Su et al. [32]
*E. coli* (XL1)	>2 log	5	Phosphate buffer	Miranda et al. [33]
*B. subtilis*	4.5 × 3 log	D/N ^1^	MOPS buffer	Qiu et al. [34]
*S. enterica*	5 log	0.5 ^2^	Food homogenates	Thavanathan et al. [35]
*E. coli* O157:H7	<10	120	Three food types ^3^	Cho and Irudayaraj [40]
*S. typhimurium*	<10	120	Three foods types ^3^	Cho and Irudayaraj [40]
*E. coli*	2 log	>180	PBS ^4^	Lim et al. [41]

^1^ D/N denotes the data not provided by the research article. ^2^ Thavanathan et al. [35] did not clarify the food types or the exact procedure to generate the food homogenates used in their experiments. ^3^ Reduced fat milk, ground beef, and pineapple juice. ^4^ Phosphate-buffered saline.

**Table 3:** Restrictions and weakness of lateral flow assays, summarized by Posthuma-Trumpie [89] and Sajid [90].

No#	Lateral Flow Assay Weaknesses
1	Difficulty of controlling capillary reaction
2	Necessity for lengthy pretreatment or cell enrichment time
3	Variation of molecular affinities and cross-reactivity
4	Imprecise sample volume reduces precision
5	Test volume restrictions limit sensitivity
6	One-step format lacks possibility to enhance the response via enzyme reaction
7	Obligatory antibody preparation or hybridization of nucleic acid sequence
8	Usually designed for individual tests, not for high-throughput screening
9	Obstruction of pores due to matrix components
10	Immunostrips usually not manufactured for this purpose
11	Obligatory sample pretreatment when the sample is not a fluid
12	Reproducibility varies
13	Response is negatively correlated to the concentration of a competitive format
14	Qualitative or semi-quantitative results

**Table 4:** Six factors worth consideration to develop the ideal pre-treatment process.

No#	Noteworthy Factors
1	The pretreated sample must be compatible with conventional or up-to-date detection methods.
2	It should be possible to shorten the total experiment time compared to the existing pre-enrichment procedure.
3	There should be little influence on the microorganisms’ growth.
4	The level of inhibitors present in the food and/or food homogenates should be minimized after pretreatment.
5	The method should ensure the effective isolation and recovery of target microorganisms from food surfaces or interiors.
6	The sample weight or buffer amount specified by regulatory organizations (e.g., ISO, USDA, and FDA) should be followed when performing the pretreatment.

## Abbreviations

FDA	Food and Drug Administration
WHO	The World Health Organization
ELISA	Enzyme-linked immunosorbent assay
SA Health	South Australian Health of Government of South Australia
CFIA	Canadian Food Inspection Agency
PFS	Public Health Surveillance Information for New Zealand Public Health Action
EFSA	European Food Safety Authority
RASFF	Rapid Alert System for Food and Feed
CDC	Center for Disease Control and Prevention
MFDS	Ministry of Food and Drug Safety of South Korea
SPR	Surface plasmon resonance
SERS	Surface enhanced Raman spectroscopy
MOPS	3-(*N*-morpholino)-propanesulfonic acid
NMR	Nuclear magnetic resonance
LOD	Limit of detection
LFA	Lateral flow chromatographic assays

## References

[1] Foodborne Illnesses: What You Need to Know. http://www.fda.gov/food/resourcesforyou/consumers/ucm103263.htm.

[2] Foodborne Germs and Illnesses. http://www.cdc.gov/foodsafety/foodborne-germs.html.

[3] Law J., Ab Mutalib N., Chan K., Lee L. (2015). Rapid Methods for The Detection of Foodborne Bacterial Pathogens: Principles, Applications, Advantages and Limitations. Front. Microbiol..

[4] Global Burden of Foodborne Diseases. http://www.who.int/foodsafety/areas_work/foodborne-diseases/ferg/en/.

[5] Clais S., Boulet G., van Kerckhoven M., Lanckacker E., Delputte P., Maes L., Cos P. (2014). Comparison of viable plate count, turbidity measurement and Real-Time PCR for quantification of *Porphyromonas Gingivalis*. Lett. Appl. Microbiol..

[6] BAM: Detection and Enumeration of *Listeria monocytogenes*. http://www.fda.gov/Food/FoodScienceResearch/LaboratoryMethods/ucm071400.htm.

[7] Government of South Australia: SA Health: Public Health Warning Issued Following Salmonella Outbreak. https://www.productsafety.gov.au/system/files/recall/SA%20Health%20Media%20Release.pdf.

[8] Canadian Food Inspection Agency: Food Recall Warning: Ajinomoto Brand Yakitori Chicken with Japanese-Style Fried Rice Recalled Due to *Listeria monocytogenes*. http://www.marketwired.com/press-release/food-recall-warning-ajinomoto-brand-yakitori-chicken-with-japanese-style-fried-rice-2123029.htm.

[9] Public Health Surveillance. https://surv.esr.cri.nz/public_health_surveillance/public_health_surveillance.php.

[10] Multi-Country Outbreak of HUS Associated with STEC Infection. http://www.efsa.europa.eu/en/supporting/pub/1017e.

[11] Rapid Alert System for Food and Feed: RASFF Portal. https://webgate.ec.europa.eu/rasff-window/portal/?event=notificationDetail&NOTIF_REFERENCE=2016.1004.

[12] Rapid Alert System for Food and Feed: RASFF Portal. https://webgate.ec.europa.eu/rasff-window/portal/?event=notificationDetail&NOTIF_REFERENCE=2016.0966.

[13] Rapid Alert System for Food and Feed: RASFF Portal. https://webgate.ec.europa.eu/rasff-window/portal/?event=notificationDetail&NOTIF_REFERENCE=2016.BAM.

[14] Rapid Alert System for Food and Feed: RASFF Portal. https://webgate.ec.europa.eu/rasff-window/portal/?event=notificationDetail&NOTIF_REFERENCE=2016.0992.

[15] Marini E., Magi G., Vincenzi C., Manso E., Facinelli B. (2016). Ongoing outbreak of invasive listeriosis due to serotype 1/2a *Listeria monocytogenes*, Ancona province, Italy, January 2015 to February 2016. Eurosurveillance.

[16] WHO: *Escherischia coli* (*E. coli*) Outbreak in United Kingdom. http://www.euro.who.int/en/health-topics/disease-prevention/food-safety/news/news/2016/07/escherichia-coli-e.-coli-outbreak-in-united-kingdom.

[17] Multistate Outbreak of Listeriosis Linked to Frozen Vegetables. http://www.cdc.gov/listeria/outbreaks/frozen-vegetables-05-16/index.html.

[18] Multistate Outbreak of Shiga toxin-producing *Escherichia coli* O121 Infections Linked to Flour. http://www.cdc.gov/ecoli/2016/o121-06-16/index.html.

[19] Multistate Outbreak of Listeriosis Linked to Raw Milk Produced by Miller’s Organic Farm in Pennsylvania. http://www.cdc.gov/listeria/outbreaks/raw-milk-03-16/index.html.

[20] Multistate Outbreak of *Salmonella* Montevideo and *Salmonella* Senftenberg Infections Linked to Wonderful Pistachios. http://www.cdc.gov/salmonella/montevideo-03-16/index.html.

[21] Multistate Outbreak of *Salmonella* Muenchen Infections Linked to Alfalfa Sprouts Produced by Sweetwater Farms. http://www.cdc.gov/salmonella/muenchen-02-16/index.html.

[22] Multistate Outbreak of Listeriosis Linked to Packaged Salads Produced at Springfield, Ohio Dole Processing Facility. http://www.cdc.gov/listeria/outbreaks/bagged-salads-01-16/index.html.

[23] Ministry of Food and Drug Safety. https://www.foodsafetykorea.go.kr/portal/healthyfoodlife/foodPoisoningStat.do?menu_no=519&menu_grp=MENU_GRP02.

[24] Fan C., Tamiya E. (2016). Editorial: Translating the Advances of Biosensors from Bench to Bedside. J. Biotechnol..

[25] Biosensors and Bioelectronics. http://www.journals.elsevier.com/biosensors-and-bioelectronics/.

[26] Lai S., O'Hanlon D., Harrold S., Man S., Wang Y., Cone R., Hanes J. (2007). Rapid Transport of Large Polymeric Nanoparticles In Fresh Undiluted Human Mucus. Proc. Natl. Acad. Sci. USA.

[27] Li F., Li Y., Feng J., Dong Y., Wang P., Chen L., Chen Z., Liu H., Wei Q. (2017). Ultrasensitive amperometric immunosensor for PSA detection based on Cu_2_O@CeO_2_-Au nanocomposites as integrated triple signal amplification strategy. Biosens. Bioelectron..

[28] Lee N., Jung Y., Park H. (2006). On-Chip Colorimetric Biosensor Based on Polydiacetylene (PDA) Embedded in Photopolymerized Poly(Ethylene Glycol) Diacrylate (PEG-DA) Hydrogel. Biochem. Eng. J..

[29] Jia H., Yang D., Han X., Cai J., Liu H., He W. (2016). Peroxidase-like activity of the Co_3_O_4_ nanoparticles used for biodetection and evaluation of antioxidant behavior. Nanoscale.

[30] He W., Zhou Y., Wamer W., Hu X., Wu X., Zheng Z., Boudreau M., Yin J. (2013). Intrinsic catalytic activity of au nanoparticles with respect to hydrogen peroxide decomposition and superoxide scavenging. Biomaterials.

[31] Liu J., Hu X., Hou S., Wen T., Liu W., Zhu X., Yin J., Wu X. (2012). Au@ Pt core/shell nanorods with peroxidase-and ascorbate oxidase-like activities for improved detection of glucose. Sens. Actuators B Chem..

[32] Su H., Zhao H., Qiao F., Chen L., Duan R., Ai S. (2013). Colorimetric Detection of *Escherichia Coli* O157:H7 using functionalized Au@Pt nanoparticles as peroxidase mimetics. Analyst.

[33] Miranda O., Li X., Garcia-Gonzalez L., Zhu Z., Yan B., Bunz U., Rotello V. (2011). Colorimetric bacteria sensing using a supramolecular enzyme–nanoparticle biosensor. J. Am. Chem. Soc..

[34] Qiu S., Lin Z., Zhou Y., Wang D., Yuan L., Wei Y., Dai T., Luo L., Chen G. (2015). Highly selective colorimetric bacteria sensing based on protein-capped nanoparticles. Analyst.

[35] Thavanathan J., Huang N., Thong K. (2015). Colorimetric Biosensing of targeted gene sequence using dual nanoparticle platforms. Int. J. Nanomed..

[36] ISO 6579:2002—Microbiology of Food and Animal Feeding Stuffs—Horizontal Method for the Detection of *Salmonella* spp.. http://www.iso.org/iso/catalogue_detail.htm?csnumber=29315.

[37] Ministry of Food and Drug Safety of South Korea. http://www.foodsafetykorea.go.kr/foodcode/menu_01_03.jsp?idx=378.

[38] WHO Global Foodborne Infections Network. http://www.antimicrobialresistance.dk/data/images/protocols/isolation_of_salm_220610.pdf.

[39] Isolation and Identification of *Salmonella* From Meat, Poultry, Pasteurized Egg, and Catfish Products and Carcass and Environmental Sponges. http://www.fsis.usda.gov/wps/wcm/connect/b0790997-2e74-48bf-9799-85814bac9ceb/28_IM_PR_Sal_Campy.pdf?MOD=AJPERES.

[40] Cho I.H., Irudayaraj J. (2013). *In-Situ* immuno-gold nanoparticle network ELISA biosensors for pathogen detection. Int. J. Food Microbiol..

[41] Lim S., Koo O., You Y., Lee Y., Kim M., Chang P., Kang D., Yu J., Choi Y., Gunasekaran S. (2012). Enhancing nanoparticle-based visible detection by controlling the extent of aggregation. Sci. Rep..

[42] Shaner N., Lin M., McKeown M., Steinbach P., Hazelwood K., Davidson M., Tsien R. (2008). Improving the photostability of bright monomeric orange and red fluorescent proteins. Nat. Methods.

[43] Dudak F., Boyacı I. (2009). Rapid and label-free bacteria detection by surface plasmon resonance (SPR) Biosensors. Biotechnol. J..

[44] Wang Y., Lee K., Irudayaraj J. (2010). Silver nanosphere SERS probes for sensitive identification of pathogens. J. Phys. Chem. C.

[45] Xu X., Chen Y., Wei H., Xia B., Liu F., Li N. (2012). Counting bacteria using functionalized gold nanoparticles as the light-scattering reporter. Anal. Chem..

[46] Phillips R., Miranda O., You C., Rotello V., Bunz U. (2008). Rapid and efficient identification of bacteria using Gold-Nanoparticle–Poly(*Para*-Phenyleneethynylene) constructs. Angew. Chem. Int. Ed..

[47] El-Boubbou K., Gruden C., Huang X. (2007). Magnetic Glyco-Nanoparticles: A Unique Tool for Rapid Pathogen Detection, Decontamination, and Strain Differentiation. J. Am. Chem. Soc..

[48] Gu H., Ho P., Tsang K., Wang L., Xu B. (2003). Using biofunctional magnetic nanoparticles to capture vancomycin-resistant enterococci and other gram-positive bacteria at ultralow concentration. J. Am. Chem. Soc..

[49] Chen L., Razavi F., Mumin A., Guo X., Sham T., Zhang J. (2013). Multifunctional nanoparticles for rapid bacterial capture, detection, and decontamination. RSC Adv..

[50] Cho I., Mauer L., Irudayaraj J. (2014). In-situ fluorescent immunomagnetic multiplex detection of foodborne pathogens in very low numbers. Biosensors and bioelectronics. Biosens. Bioelectron..

[51] Kaittanis C., Naser S., Perez J. (2007). One-step, nanoparticle-mediated bacterial detection with magnetic relaxation. Nano Lett..

[52] Lee H., Yoon T., Weissleder R. (2009). Ultrasensitive detection of bacteria using core-shell nanoparticles and an NMR-filter system. Angew. Chem..

[53] Ricci F., Adornetto G., Palleschi G. (2012). A review of experimental aspects of electrochemical immunosensors. Electrochim. Acta.

[54] Doria G., Conde J., Veigas B., Giestas L., Almeida C., Assunção M., Rosa J., Baptista P. (2012). Noble metal nanoparticles for biosensing applications. Sensors.

[55] Varshney M., Li Y. (2007). Interdigitated Array Microelectrode based Impedance Biosensor Coupled with Magnetic Nanoparticle–Antibody Conjugates for Detection of Escherichia Coli O157:H7 in Food Samples. Biosens. Bioelectron..

[56] CDC Online Newsroom. https://www.cdc.gov/media/releases/2013/p0516-pool-contamination.html.

[57] BAM: Diarrheagenic *Escherichia coli*. http://www.fda.gov/Food/FoodScienceResearch/LaboratoryMethods/ucm070080.htm.

[58] Xu D., Watt G., Harb J., Davis R. (2005). Electrical conductivity of ferritin proteins by conductive AFM. Nano Lett..

[59] Zhang X., Shao J., Jiang S., Wang B., Zheng Y. (2015). Structure-dependent electrical conductivity of protein: Its differences between α-domain and β-domain structures. Nanotechnology.

[60] Setterington E., Alocilja E. (2012). Electrochemical biosensor for rapid and sensitive detection of magnetically extracted bacterial pathogens. Biosensors.

[61] Afonso A., Pérez-López B., Faria R., Mattoso L., Hernández-Herrero M., Roig-Sagués A., Maltez-da Costa M., Merkoçi A. (2013). Electrochemical detection of *Salmonella* using gold nanoparticles. Biosens. Bioelectron..

[62] Wang H., Zhang Y., Wang Y., Ma H., Du B., Wei Q. (2017). Facile synthesis of cuprous oxide nanowires decorated graphene oxide nanosheets nanocomposites and its application in label-free electrochemical immunosensor. Biosens. Bioelectron..

[63] Chen J., Andler S.M., Goddard J.M., Nugen S.R., Rotello V.M. (2017). Integrating recognition elements with nanomaterials for bacteria sensing. Chem. Soc. Rev..

[64] Sanchez-Tirado E., Gonzalez-Cortes A., Yanez-Sedeno P., Pingarron J.M. (2016). Carbon nanotubes functionalized by click chemistry as scaffolds for the preparation of electrochemical immunosensors. Application to the determination of TGF-β 1 cytokine. Analyst.

[65] Tlili C., Cella L.N., Myung N.V., Shetty V., Mulchandani A. (2010). Single-walled carbon nanotube chemoresistive label-free immunosensor for salivary stress biomarkers. Analyst.

[66] Kuila T., Bose S., Khanra P., Mishra A.K., Kim N.H., Lee J.H. (2011). Recent advances in graphene-based biosensors. Biosens. Bioelectron..

[67] Cao X., Liu S., Feng Q., Wang N. (2013). Silver nanowire-based electrochemical immunoassay for sensing immunoglobulin G with signal amplification using strawberry-like ZnO nanostructures as labels. Biosens. Bioelectron..

[68] He B., Morrow T.J., Keating C.D. (2008). Nanowire sensors for multiplexed detection of biomolecules. Curr. Opin. Chem. Biol..

[69] Craig A., Franca A., Irudayaraj J. (2013). Surface-enhanced Raman spectroscopy applied to food safety. Annu. Rev. Food Sci. Technol..

[70] Popp J. (2007). Identification of micro-organisms by Raman spectroscopy. Spie Newsroom..

[71] Pahlow S., Meisel S., Cialla-May D., Weber K., Rösch P., Popp J. (2015). Isolation and identification of bacteria by means of Raman spectroscopy. Adv. Drug Deliv. Rev..

[72] Sattler K. (2011). Handbook of Nanophysics: Nanoelectronics and Nanophotonics.

[73] Ravindranath S., Wang Y., Irudayaraj J. (2011). SERS driven cross-platform based multiplex pathogen detection. Sens. Actuators B Chem..

[74] Singhal N., Kumar M., Kanaujia P., Virdi J. (2015). MALDI-TOF mass spectrometry: An emerging technology for microbial identification and diagnosis. Front. Microbiol..

[75] Seng P., Drancourt M., Gouriet F., La Scola B., Fournier P., Rolain J., Raoult D. (2009). ongoing revolution in bacteriology: Routine identification of bacteria by matrix-assisted laser desorption ionization time-of-flight mass spectrometry. Clin. Infect. Dis..

[76] Chiang C., Chiang M., Lin Z., Lan G., Lin Y., Chang H. (2010). Nanomaterial-based surface-assisted laser desorption/ionization mass spectrometry of peptides and proteins. J. Am. Soc. Mass Spectrom..

[77] Ahmad F., Siddiqui M., Babalola O., Wu H. (2012). Biofunctionalization of nanoparticle assisted mass spectrometry as biosensors for rapid detection of plant associated bacteria. Biosens. Bioelectron..

[78] Cho I., Paek E., Kim Y., Kim J., Paek S. (2009). Chemiluminometric enzyme-linked immunosorbent assays (ELISA)-on-a-chip biosensor based on cross-flow chromatography. Anal. Chim. Acta.

[79] Cho I., Radadia A., Farrokhzad K., Ximenes E., Bae E., Singh A., Oliver H., Ladisch M., Bhunia A., Applegate B. (2014). Nano/Micro and spectroscopic approaches to food pathogen detection. Annu. Rev. Anal. Chem..

[80] Cho I., Bhunia A., Irudayaraj J. (2015). Rapid pathogen detection by lateral-flow immunochromatographic assay with gold nanoparticle-assisted enzyme signal amplification. Int. J. Food Microbiol..

[81] Li X., Luo P., Tang S., Beier R.C., Wu X., Yang L., Li Y., Xiao X. (2011). Development of an immunochromatographic strip test for rapid detection of melamine in raw milk, milk products and animal feed. J. Agric. Food Chem..

[82] Liu L.Q., Luo L.J., Suryoprabowo S., Peng J., Kuang H., Xu C.L. (2014). Development of an immunochromatographic strip test for rapid detection of ciprofloxacin in milk samples. Sensors.

[83] Zhang X., Wu C., Wen K., Jiang H., Shen J., Zhang S., Wang Z. (2016). Comparison of fluorescent microspheres and colloidal gold as labels in lateral flow immunochromatographic assays for the detection of T-2 toxin. Molecules.

[84] Pengsuk C., Chaivisuthangkura P., Longyant S., Sithigorngul P. (2013). Development and evaluation of a highly sensitive immunochromatographic strip test using gold nanoparticle for direct detection of vibrio cholerae O139 in seafood samples. Biosens. Bioelectron..

[85] Cho I., Irudayaraj J. (2013). Lateral-flow enzyme immunoconcentration for rapid detection of listeria monocytogenes. Anal. Bioanal. Chem..

[86] Ho J., Zeng S., Tseng W., Lin Y., Chen C. (2008). Liposome-based immunostrip for the rapid detection of *Salmonella*. Anal. Bioanal. Chem..

[87] Shukla S., Leem H., Kim M. (2011). Development of a liposome-based immunochromatographic strip assay for the detection of *Salmonella*. Anal. Bioanal. Chem..

[88] Lutz B., Liang T., Fu E., Ramachandran S., Kauffman P., Yager P. (2013). Dissolvable fluidic time delays for programming multi-step assays in instrument-free paper diagnostics. Lab Chip.

[89] Posthuma-Trumpie G., Korf J., van Amerongen A. (2008). lateral flow (immuno) assay: Its strengths, weaknesses, opportunities and threats. A literature survey. Anal. Bioanal. Chem..

[90] Sajid M., Kawde A., Daud M. (2015). Designs, Formats and applications of lateral flow assay: A literature review. J. Saudi Chem. Soc..

[91] Li X. (2014). Improved Detection Techniques for Foodborne Pathogens: Separation Techniques Using Crossflow Microfiltration. Ph.D. Thesis.

[92] Ku S. (2015). Rapid *Salmonella* Concentration, Recovery and Detection from Food Samples. Ph.D. Thesis.

[93] FDA Food Safety Challenge Winners Develop Innovative Technologies to Detect *Salmonella*. https://www.fda.gov/NewsEvents/Newsroom/PressAnnouncements/ucm455660.htm.

[94] Ku S., Ximenes E., Kreke T., Foster K., Deering A., Ladisch M. (2016). Microfiltration of enzyme treated egg whites for accelerated detection of viable *Salmonella*. Biotechnol. Prog..

[95] Ku S., Ximenes E., Kreke T., Ladisch M. (2016). *Salmonella* in shell eggs: Mechanisms, prevention and detection. J. Nutr. Food Sci..

[96] Ku S., Kreke T., Ximenes E., Foster K., Liu X., Gilpin C., Ladisch M. (2017). Protein particulate retention and microorganism recovery for rapid detection of *Salmonella*. Biotechnol. Prog..

[97] Li X., Ximenes E., Amalaradjou M., Vibbert H., Foster K., Jones J., Liu X., Bhunia A., Ladisch M. (2013). Rapid sample processing for detection of food-borne pathogens via cross-flow microfiltration. Appl. Environ. Microbiol..

[98] Vibbert H., Ku S., Li X., Liu X., Ximenes E., Kreke T., Ladisch M., Deering A., Gehring A. (2015). Accelerating sample preparation through enzyme-assisted microfiltration of *Salmonella* in chicken extract. Biotechnol. Prog..

[99] Ximenes E., Hoagland L., Ku S., Li X., Ladisch M. (2017). Human pathogens in plant biofilms: Formation, physiology, and detection. Biotechnol. Bioeng..

